# The Chinese species of the genus *Ontsira* Cameron (Hymenoptera, Braconidae, Doryctinae)

**DOI:** 10.3897/zookeys.345.5472

**Published:** 2013-10-29

**Authors:** Sergey A. Belokobylskij, Pu Tang, Xue-xin Chen

**Affiliations:** 1 Zoological Institute Russian Academy of Sciences, St. Petersburg, 199034, Russia; Museum and Institute of Zoology Polish Academy of Sciences, Wilcza 64, Warszawa 00–679, Poland; 2 State Key Laboratory of Rice Biology and Ministry of Agriculture Key Lab of Agricultural Entomology, Institute of Insect Sciences, Zhejiang University, Hangzhou 310058, China

**Keywords:** Ectoparasitoid, Braconidae, Doryctinae, *Ontsira*, new species, new record, key, Asia

## Abstract

The Chinese species of the genus *Ontsira* Cameron, 1900 (Hymenoptera: Braconidae, Doryctinae) are reviewed. Eleven species are recognized, of which four new species are described from China and South Korea: *O. abbreviata* **sp. n.**, *O. henana* **sp. n.**, *O. robusta* **sp. n.**, and *O. rugivertex* **sp. n.** Two species, *O. ignea* (Ratzeburg) and *O. neantica* Belokobylskij et Maetô, are recorded in China for the first time. A key to the Asian species of the genus *Ontsira* is provided.

## Introduction

The genus *Ontsira* Cameron, 1900 is not a species-rich genus and is one of the less specialised taxa of the large tribe Doryctini (Hymenoptera: Braconidae, Doryctinae) (Belokobylskij 1992, 1998a). Twenty-nine species are known, including the new taxa described here. They are distributed in the Holarctic (18 species), Oriental (9 species), Australasian (1 species) and Afrotropical (1 species) regions, but no species have yet been found in the Neotropics. Members of *Ontsira* are known as ectoparasitoids of mainly xylophagous beetle larvae of the families Anobiidae, Bostrichidae, Buprestidae, Cerambycidae (prevalently), Curculionidae, Scolytidae, Eucnemidae, Nitidulidae and Tenebrionidae (Shenefelt and Marsh 1976; Belokobylskij and Maetô 2009; Yu et al. 2012), but the records of the last three families need confirmation.

After recent morphological and molecular studies (Belokobylskij 1998a, 2008; Zaldivar-Riverón et al. 2008; Belokobylskij and Maetô 2009), some species formerly belonging to *Ontsira* have been moved to other existing genera, such as *Dolopsidea* Hincks, 1944 and *Neurocrassus* Šnoflak, 1940 (Belokobylskij 1982, 1998a; Belokobylskij and Maetô 2009) or to new genera established by Belokobylskij (1998b, 2008), such as *Rhacontsira* Belokobylskij, 1998 and *Cryptontsira* Belokobylskij, 2008.

Only three species of this genus were previously described from China (Chen and Shi 2004). However, according to Belokobylskij and Maetô (2009), *Ontsira brachytes* Chen & Shi, 2004 is a junior synonym of *Ontsira apposita* Belokobylskij, 1998 and *Ontsira retina* Chen & Shi, 2004 a junior synonym of *Hypodoryctes fuga* Belokobylskij & Chen, 2004. In this study, another four new species of *Ontsira* are described from China and South Korea, and two species, *Ontsira ignea* (Ratzeburg, 1852) and *Ontsira neantica* Belokobylskij & Maetô, 2009, are recorded for the fauna of China for the first time.

## Material and methods

This study is based on specimens from the Parasitic Hymenoptera Collection of the Institute of Insect Sciences, Zhejiang University, Hangzhou, China (ZJUH) and Zoological Institute of the Russian Academy of Sciences, St. Petersburg, Russia (ZISP); material from other museums was not available in the period of our investigation. The terms of wing venation are used as defined by Belokobylskij and Maetô (2009). The following abbreviations are used: POL – postocellar line; OOL – ocular-ocellar line; Od – maximum diameter of lateral ocellus. In the key, additional features useful for separating species are listed after the dash “–”.

The descriptions and measurements were made under a Micromed MC–2 ZOOM stereomicroscope, and all figures were made by a digital camera (Q-Iming, Micropublisher, 3.3 RTV) attached to a stereomicroscope (Leica MZ APO, Germany) and Auto-Montage Pro version 5.0 software. Type specimens and other materials are deposited in the collection of ZISP and ZJUH.

## Taxonomic part

### Genus Ontsira

Cameron, 1900

http://species-id.net/wiki/Ontsira

Ontsira Cameron, 1900: 89; Shenefelt and Marsh 1976: 1322; Belokobylskij and Tobias 1986: 41; Belokobylskij 1998a: 464; 1998c: 54; Chen and Shi 2004: 27; Belokobylskij and Maetô 2009: 363.
Doryctes (Doryctodes) Hellen, 1927: 40 [type species: *Rogas (Doryctes) imperator* Haliday, 1836]; Shenefelt and Marsh 1976: 1322.
Doryctodes : Telenga, 1941: 389; Shenefelt and Marsh 1976: 1322.
Wachsmannia Szépligeti, 1900: 217 (type species: *Wachsmannia maculipennis* Szépligeti, 1900); Shenefelt and Marsh 1976: 1332; Belokobylskij 1992: 908; van Achterberg 1995: 131; Belokobylskij 1998a: 462.

#### Type species.

*Ontsira reticulata* Cameron, 1900.

#### Key to the Asian species of the genus *Ontsira* Cameron

(Update from Belokobylskij and Maetô 2009)

**Table:** 

1	Second tergite with more or less smooth basomedian area separated by furrow or different type of sculpture. Body usually with contrasted pale and dark colouration. Upper tentorial pits (latero-posteriorly from antennal sockets) present, small or very small	*Neurocrassus* Šnoflak
–	Second tergite without smooth basomedian area. Body usually without contrasted colouration. Upper tentorial pits completely absent	2
2	Second metasomal tergite completely smooth	3
–	Second metasomal tergite sculptured at least basally	7
3	First metasomal tergite shorter, not longer than its apical width. Mesoscutum mostly smooth or finely granulate in anterior part	4
–	First metasomal tergite longer, 1.15–1.30 times as long as its apical width. Mesoscutum entirely and distinctly granulate	5
4	Ovipositor shorter, its sheath 0.5–0.7 times as long as metasoma, 0.65–0.80 times as long as mesosoma, 0.30–0.35 times as long as fore wing. Transverse diameter of eye 1.0–1.1 times as long as temple. Propodeum with distinct lateral tubercles. First metasomal tergite shorter, 0.8–0.9 times as long as its apical width. Body length 3.0–4.5 mm. – China (Heilongjiang); Russia, Caucasus, Turkey, Central and West Europe, USA	*Ontsira antica* (Wollaston)
–	Ovipositor longer, its sheath 0.9–1.0 times as long as metasoma, distinctly longer than mesosoma, about 0.5–0.6 times as long as fore wing. Transverse diameter of eye 0.9 times as long as temple. Propodeum almost without lateral tubercles. First metasomal tergite longer, its length equal to its apical width. Body length 3.1–5.3 mm. – China (Heilongjiang); Japan	*Ontsira neantica* Belokobylskij & Maetô
5	Parallel vein arising almost from middle of distal margin of brachial cell. Second radial abscissa 0.8 times as long as third abscissa. Second radiomedial cell 2.7 times as long as its maximum width. Basolateral areas of propodeum granulate. Median length of second tergite 1.4 times as long as median length of third tergite. – First tergite entirely striate. Nervulus of fore wing strongly postfurcal. Ovipositor sheath 0.4 times as long as fore wing. Body length 3.6 mm. – China (Taiwan)	*Ontsira gratia* Belokobylskij
–	Parallel vein arising from posterior 0.25–0.30 of distal margin of brachial cell. Second radial abscissa 0.50–0.65 times as long as third abscissa. Second radiomedial cell 2.3–2.4 times as long as its maximum width. Basolateral areas of propodeum entirely or mainly smooth, sometimes rugulose posteriorly. Median length of second tergite almost equal to median length of third tergite	6
6	Nervulus of fore wing almost interstitial. Ovipositor sheath 0.7 times as long as fore wing. Hind coxa smooth dorsally. First metasomal tergite longer, 1.25 times as long as its apical width. Hind coxa and femur mainly dark reddish brown. Frons without pit between antennal sockets. Body length 5.6 mm. – India	*Ontsira reticulata* Cameron
–	Nervulus of fore wing distinctly postfurcal. Ovipositor sheath 0.4 times as long as fore wing. Hind coxa distinctly sculptured dorsally. First metasomal tergite shorter, 1.15 times as long as its apical width. Hind coxa and femur entirely yellow. Frons with distinct elongate pit between antennal sockets. Body length 3.0 mm. – China (Shaanxi)	*Ontsira abbreviata* sp. n.
7	Second tergite sculptured only basally or in basal half	8
–	Second tergite entirely or almost entirely sculptured, but sometimes sculpture in apical half fine and interrupted	17
8	Temple very short, transverse diameter of eye 3.3–3.8 times as long as temple. Acrosternite of first tergite rather distinctly elongate. Apical segments of antenna white. Body length 3.0–3.7 mm. – China (Fujian, Zhejiang); Vietnam	*Ontsira apposita* Belokobylskij
–	Temple longer, transverse diameter of eye 1.0–1.6 times as long as temple. Acrosternite of first tergite shorter. Apical segments of antenna dark	9
9	Mesoscutum and basolateral areas of propodeum completely smooth. Mesosoma 1.5 times as long as its maximum height. Eyes glabrous. Radial vein arising slightly before middle of pterostigma. Body length 2.1 mm. – Vietnam	*Ontsira tayi* Belokobylskij
–	Mesoscutum and basolateral areas of propodeum more or less granulate or sometimes finely granulate and with distinct punctation. Mesosoma 1.7–2.0 times as long as its maximum height. Eyes often (except *Ontsira henana* sp. n.) rather densely and shortly setose. Radial vein arising more or less behind middle of pterostigma or sometimes from its middle	10
10	Ovipositor sheath not longer than metasoma. Mesoscutum and scutellum distinctly and densely granulate. Parallel vein of fore wing arising slightly before or almost from middle of distal margin of brachial cell. First abscissa of mediocubital vein of hind wing almost equal to second abscissa	11
–	Ovipositor sheath longer than metasoma, almost as long as body. Mesoscutum finely granulate; scutellum almost smooth. Parallel vein of fore wing arising from posterior 0.25–0.30 of distal margin of brachial cell. First abscissa of mediocubital vein of hind wing longer than second abscissa	12
11	Vertex almost entirely and finely granulate-reticulate; frons at least partly rugulose-granulate. Eyes distinctly setose. Antennal segments thick, first flagellar segment 3.4–3.5 times as long as its apical width. Third tergite without transverse furrow. Vertex and mesoscutum with long setae. Body length 2.8–3.1 mm. – Russia (Far East)	*Ontsira eugeniae* Belokobylskij
–	Vertex and most part of frons smooth. Eyes glabrous. Antennal segments slender, first flagellar segment 4.3 times as long as its apical width. Third tergite with transverse furrow. Vertex and mesoscutum with short setae. Body length 2.8 mm. – China (Henan)	*Ontsira henana* sp. n.
12	First metasomal tergite shorter, 0.9–1.1 (rarely 1.2) times as long as its apical width. Hind femur wide, 3.4–4.0 times as long as wide. Penultimate segment 1.7–2.0 times as long as wide	13
–	First metasomal tergite longer, 1.2–1.7 times as long as its apical width. Hind femur slender, 4.0–5.3 times as long as wide. Penultimate segment 2.1–2.4 times as long as wide	14
13	Vertex entirely or mostly and temple almost entirely smooth. Mesoscutum (except rugose-striate in medioposterior area) mainly smooth, partly finely granulate. Body length 2.5–7.5 mm. – China (Taiwan); Japan, Korea, Mongolia, Russia, Kazakhstan, Iran, Caucasus, Central and Western Europe, North America	*Ontsira imperator* (Haliday)
–	Vertex almost entirely or mostly and temple at most part distinctly striate. Mesoscutum (except rugose-striate in medioposterior area) almost entirely, very densely and finely granulate with sparse punctation. Body length 6.0–7.2 mm. – China (North-east part); South Korea	*Ontsira robusta* sp. n.
14	Hind femur 4.0–4.5 times as long as its maximum width. First metasomal tergite 1.2–1.3 (rarely 1.15 or 1.40) times as long as its apical width. Second metasomal tergite shorter, 0.5–0.8 times as long as its basal width. Hind tibia dorsally with very dense setae	15
–	Hind femur 4.9–5.3 times as long as its maximum width. First metasomal tergite 1.4–1.7 times as long as its apical width. Second metasomal tergite longer, 0.9 times as long as its basal width. Hind tibia dorsally with sparse setae	16
15	Vertex entirely smooth. Mesoscutum rather finely punctate-reticulate, partly with fine granulation. Second tarsal segment of hind leg shorter, 1.4–1.5 times as long as its fifth segment (without pretarsus) and about 0.4 times as long as basitarsus. First flagellar segment longer, 4.0–4.5 times as long as wide. Body length 5.0–7.4 mm. – China (Shaanxi, Fujian, Guangdong); Japan, Korea, Russia, Caucasus, Turkey, Israel, Europe	*Ontsira ignea* (Ratzeburg)
–	Vertex entirely or almost entirely coarsely rugose-striate. Mesoscutum coarsely rugose-granulate. Second tarsal segment of hind leg longer, 1.5–1.7 times as long as its fifth segment (without pretarsus) and about 0.5 times as long as basitarsus. First flagellar segment shorter, 3.4–3.6 times as long as wide. Body length 5.5–7.0 mm. – China (Henan, Shaanxi)	*Ontsira rugivertex* sp. n.
16	Brachial cell of fore wing distinctly widened medially. Basolateral areas of propodeum without carinae. Pterostigma and radial cell longer; pterostigma 4.8 times and radial cell 3.9–4.0 times as long as their maximum width. Body length 7.5 mm. – Japan	*Ontsira amamioshima* Belokobylskij & Maetô
–	Brachial cell of fore wing not widened medially. Basolateral areas of propodeum with distinct carinae. Pterostigma and radial cell shorter; pterostigma 4.4–4.6 times and radial cell 3.4–3.6 times as long as their maximum width. Body length 5.4–8.4 mm. – China (Zhejiang, Fujian, Guangdong)	*Ontsira macer* Chen & Shi
17(7)	Antennae 17–19-segmented. Female body length 2.5–3.0 mm	18
–	Antennae 30–40-segmented. Female body length 4.0–7.0 mm	19
18	Recurrent vein of fore wing almost as long as second abscissa of medial vein. Antenna 17-segmented. Head pale brown, mesosoma and metasoma dark brown. Body length 2.5 mm. – Vietnam	*Ontsira bistriata* (Kieffer)
–	Recurrent vein of fore wing 0.3–0.5 times as long as second abscissa of medial vein. Antenna 19-segmented. Head and all body brownish yellow. Body length 3.0 mm. – Vietnam	*Ontsira brevipetiolata* (Kieffer)
19	Ovipositor sheath 0.8 times as long as metasoma. Third metasomal tergite submedially with transverse furrow. Transverse diameter of eye 2.8 times as long as temple. Seven apical segments of antenna white. Parallel vein of fore wing arising weakly behind middle of distal margin of brachial cell. Body length 3.3 mm. – Vietnam	*Ontsira alboapicalis* Belokobylskij
–	Ovipositor sheath much longer than metasoma. Third metasomal tergite without transverse sculptured furrow. Transverse diameter of eye 1.0–1.3 times as long as temple. Apical segments of antenna dark. Parallel vein of fore wing arising strongly behind middle of distal margin of brachial cell	20
20	Third metasomal tergite entirely smooth. Second tergite sometimes finely and interruptedly striate in posterior 0.3. Body length 5.0–7.5 mm. – Japan	*Ontsira ignea* (Ratzeburg) (f. *insularis* Belokobylskij)
–	Third metasomal tergite basally with semi-circular striae. Second tergite always completely striate. Body length 4.0–6.2 mm. – Russia (Far East)	*Ontsira kasparyani* Belokobylskij

### Review of Chinese species

#### Ontsira abbreviata sp. n.

http://zoobank.org/C79C62F9-32ED-4F77-8457-4B7D1142975D

http://species-id.net/wiki/Ontsira_abbreviata

Fig. 1

##### Type material.

Holotype: female, China, Shaanxi, Huoditang, 5.VI.1998 (Du Yuzhou), N 982455 (ZJUH).

##### Etymology.

From Latin “abbreviatae”, meaning “shortened”, after the shortened second radiomedial cell of the fore wing

##### Description.

Female. Body length 3.0 mm; fore wing length 2.7 mm.

Head width 1.6 times as long as its median length, 1.2 times as long as width of mesoscutum. Frons without carina, with distinct elongate pit between antennal sockets. Head behind eyes (dorsal view) regularly roundly narrowed; transverse diameter of eye 1.2 times as long as temple. Ocellar triangle situated behind middle of head (dorsal view), its anterior ocellus situated almost on level of anterior margins of eyes. Ocelli small, in almost equilateral triangle. POL 1.2 times as long as Od, 0.3 times as long as OOL. Eye sparsely and shortly setose, without emargination opposite antennal sockets, 1.2 times as high as broad. Face along eyes without carinae, with small shallow depressions above clypeus; width of face 1.4 times height of eye and 1.4 times height of face and clypeus combined. Diameter of antennal socket almost equal to distance between sockets and distance between socket and eye. Malar suture indistinct. Malar space about 0.6 times height of eye and 0.9 times basal width of mandible. Clypeus with distinct lower flange. Clypeal suture distinct and complete. Hypoclypeal depression oval, its width almost equal to distance from edge of depression to eye, 0.4 times width of face. Occipital carina ventrally fused with hypostomal carina at upper base of mandible. Maxillary palpi long, 1.5 times as long as head height.

Antennae rather slender, filiform, 25-segmented, as long as body. Scape 1.9 times as long as its maximum width. First flagellar segment 5.0 times as long as its apical width, and 1.4 times as long as second segment. Penultimate segment 2.7 times as long as wide, 0.5 times as long as first flagellar segment, and 0.9 times as long as apical segment; the apical segment distinctly pointed apically.

Mesosoma. Length 1.8 times its height. Pronotum not convex dorsally (lateral view), with distinct pronotal carina. Median lobe of mesoscutum (dorsal view) weakly protruding forward, with wide median furrow. Notauli deep, narrow, crenulate-rugulose. Prescutellar depression rather deep, with three median carinae, finely rugulose to smooth between carinae, 0.4 times as long as scutellum. Scutellum convex, without lateral carinae. Metanotum (dorsal view) with two strongly convergent and fused posteriorly lateral carinae, without median carinae; with short and obtuse metanotal tooth. Subalar depression rather shallow, wide, sparsely rugose-striate. Sternaulus deep, straight, finely crenulate, connected with prepectal carina anteriorly, running along anterior 0.6 of the lower part of mesopleuron. Metapleural flange long, wide, rounded apically. Propodeum with short, thick lateral tubercles.

Wings. Fore wing 3.1 times as long as maximum width. Radial vein arising weakly behind middle of pterostigma, from its basal 0.55. Radial cell not shortened; metacarp 1.25 times as long as pterostigma. First radial abscissa 0.5 times as long as maximum width of pterostigma. Second radial abscissa 4.5 times as long as first abscissa, 0.5 times as long as the straight third abscissa, and 1.15 times as long as first radiomedial vein. Second radiomedial cell 2.4 times as long as its maximum width, and 1.7 times as long as brachial cell. First medial abscissa weakly sinuate. Mediocubital vein almost straight. Recurrent vein distinctly antefurcal. Distance from nervulus to basal vein about 0.5 times as long as nervulus. Parallel vein arising distinctly behind middle of distal margin of brachial cell. Hind wing 4.5 times as long as maximum width. First costal abscissa 0.7 times as long as second abscissa. First abscissa of mediocubital vein 1.1 times as long as second abscissa. Mediocubital cell large, weakly widened toward apex, 7.0 times as long as wide, and 0.4 times as long as wing. Recurrent vein weakly curved, strongly antefurcal, unsclerotized.

Legs. Fore tibia with rather numerous slender spines arranged in almost single line. Hind coxa without dorsal tooth, 1.6 times as long as wide. Hind femur 4.2 times as long as wide. Hind tarsus 0.9 times as long as hind tibia. Hind basitarsus 0.7 times as long as second-fifth segments combined. Second segment of hind tarsus 0.45 times as long as basitarsus, 1.1 times as long as fifth segment (without pretarsus).

Metasoma about as long as head and mesosoma combined. First tergite with distinct dorsope, with small spiracular tubercles in basal 0.3, rather distinctly and almost linearly widened from base to apex. Maximum width of first tergite about twice its minimum width; length 1.15 times as long as its apical width. Second tergite without basomedian area; length of tergite 0.7 times as long as its basal width, almost equal to length of third tergite. Second suture very shallow and straight. Third tergite without additional transverse furrow. Ovipositor sheath 0.8 times as long as metasoma, as long as mesosoma, and 0.4 times as long as fore wing.

Sculpture and pubescence. Vertex and temple smooth; frons mostly smooth, finely coriaceous anterolaterally; face mostly smooth, finely rugulose-striate on upper part and on clypeus. Sides of pronotum smooth ventrally, coarsely crenulate medially, rugulose dorsally. Mesoscutum densely and distinctly granulate, rugulose anteriorly. Scutellum entirely finely granulate. Mesopleuron mostly smooth. Propodeum with distinctly areas delineated by carinae; basolateral areas large, smooth, but rugose in posterior 0.3 and along carinae; areola long and narrow, twice as long as wide; dorsal carina rather long, about twice as long as areola fork. Hind coxae coarsely striate in dorsal half, finely rugulose to smooth in ventral half. Hind femur smooth. First tergite with high complete subparallel dorsal carinae, rather distinctly striate, but smooth mediobasally (between dorsal carinae) and lateroapically. Second and the following tergites smooth. Vertex with rather sparse, long and semi-erect setae, glabrous in anterior half. Mesoscutum with dense, rather long and semi-erect pale setae, glabrous in rather wide submedian areas on lateral lobes. Hind tibia dorsally with rather long, sparse semi-erect setae basally and densely apically, length of these setae 0.7–1.0 times as long as maximum width of hind tibia.

Colour. Body dark reddish brown, metasoma behind first tergite reddish brown, malar area of head yellowish brown. Antennae dark brown, light reddish brown basally. Palpi yellow. Legs entirely yellow. Ovipositor sheath black. Forewing membrane faintly infuscate. Pterostigma brown, paler basally and apically.

Male unknown.

**Figure 1.**
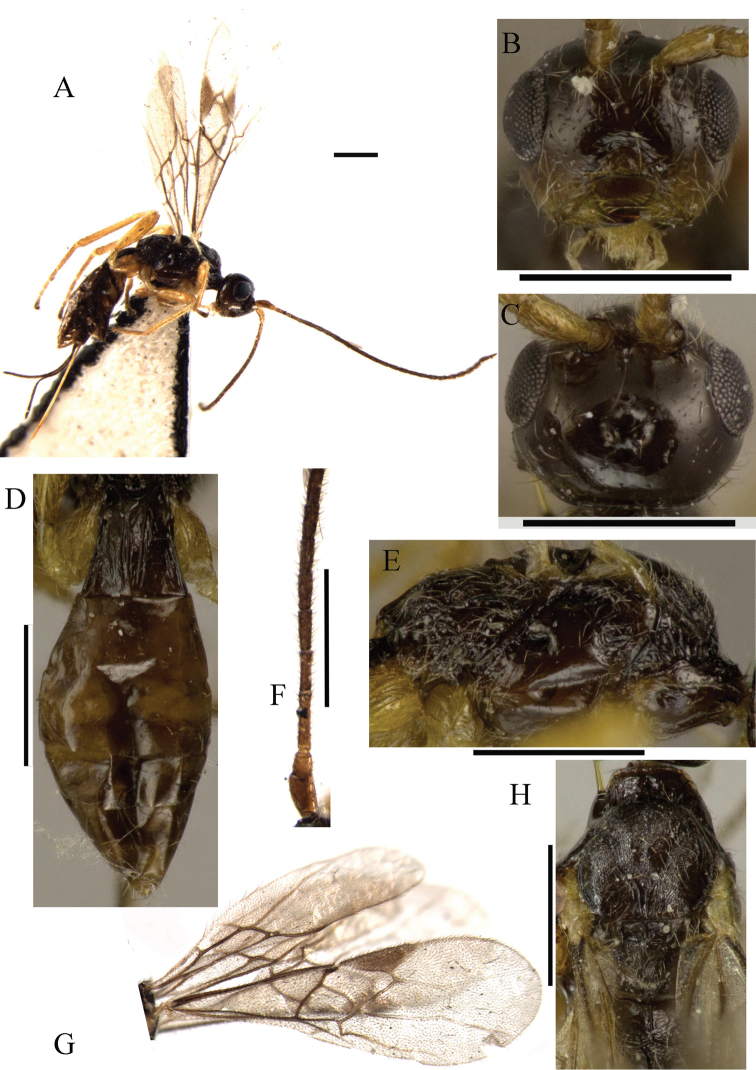
*Ontsira abbreviata* sp. n. (female). **A** habitus, lateral view **B** head, front view **C** head, dorsal view **D** metasoma, dorsal view **E** mesosoma, lateral view **F** basal segments of antenna **G** fore and hind wings **H** mesosoma, dorsal view. Scale bar 0.5 mm.

##### Distribution.

China (Shaanxi).

##### Diagnosis.

The new species is similar to *Ontsira reticulata* Cameron (India), but differs in having the second radiomedial cell and the second radial abscissa short, the nervulus postfurcal, the ovipositor shorter, the hind coxa sculptured dorsally, the legs entirely yellow, the first tergite shorter, and the frons with distinct elongate pit between antennal sockets. It also resembles *Ontsira gratia* Belokobylskij (from Taiwan), but differ in having the parallel vein arising distinctly behind middle of distal margin of brachial cell, the occipital carina fused below with hypostomal carina, the basolateral areas of propodeum without granulation, the radial vein arising weakly behind middle of pterostigma, and the second radial abscissa and the second radiomedial cell shorter.

#### Ontsira antica

(Wollaston, 1858)

http://species-id.net/wiki/Ontsira_antica

Clinocentrus anticus Wollaston, 1858: 24; Yu et al. 2012.
Ontsira antica : Marsh, 1973: 71; Shenefelt and Marsh 1976: 1322.
Doryctes gallicus Reinhard, 1865: 248; Shenefelt and Marsh 1976: 1322; Yu et al. 2012.

##### Material examined.

China: 1 female, Heilongjiang, Daiceng, 24.VII.1977 (He Junhua), N 771882 (ZJUH).

##### Distribution.

China (Heilongjiang) (**new record**); Russia, Caucasus, Central and Western Europe, North America.

#### Ontsira apposita

Belokobylskij, 1998

http://species-id.net/wiki/Ontsira_apposita

Ontsira apposita Belokobylskij, 1998: 466; Yu et al. 2012.
Ontsira brachytes Chen & Shi, 2004: 27; Belokobylskij and Maetô 2009: 363.

##### Material examined.

China: 1 male, Zhejiang, Gutianshan, 19.VII.1992 (Chen Xuexin), N 923661 (ZJUH).

##### Distribution.

China (Zhejiang, Fujian); Vietnam.

##### Remark.

The male of *Ontsira apposita* is recorded for the first time. It differs from females in the following characters: the parallel vein of the fore wing arises slightly behind middle of distal margin of brachial cell, the recurrent vein of fore wing antefurcal, the first abscissa of mediocubital vein of hind wing not shorter than second abscissa, and the acrosternite of first metasomal segment weakly elongate.

#### Ontsira henana sp. n.

http://zoobank.org/E6BFCC07-1F4E-435B-876F-4B53E4752EF9

http://species-id.net/wiki/Ontsira_henana

Fig. 2

##### Type material.

Holotype: female, China, Henan, Neixiang, Baotianman, 15.VII.1998 (Chen Xuexin), N 989014 (ZJUH).

##### Etymology.

Named after the locality of the holotype, Henan province.

##### Description.

Female. Body length 2.8 mm; fore wing length 2.7 mm.

Head width 1.5 times as long as its median length, 1.15 times as long as width of mesoscutum. Frons without carina, with very shallow median furrow. Head behind eyes (dorsal view) regularly roundly narrowed; transverse diameter of eye 1.6 times as long as temple. Ocellar triangle situated on middle of head (dorsal view), its anterior ocellus situated weakly behind middle level of eyes. Ocelli small, in almost equilateral triangle. POL 1.3 times as long as Od, 0.4 times as long as OOL. Eye glabrous, without emargination opposite antennal socket, 1.1 times as high as broad. Face along eyes without carinae, with small shallow elongate depressions above clypeus; width of face 1.4 times as long as height of eye and 1.4 times as long as height of face and clypeus combined. Diameter of antennal socket 1.4 times as long as distance between sockets and 1.4 times as long as distance between socket and eye. Malar suture indistinct. Malar space 0.5 times as long as height of eye and equal to basal width of mandible. Clypeus with distinct lower flange. Clypeal suture distinct and complete. Hypoclypeal depression round, its width 0.8 times as long as distance from edge of depression to eye and 0.35 times as long as width of face. Occipital carina ventrally not fused with hypostomal carina. Maxillary palpi long, 1.4 times as long as head height.

Antennae rather thick, almost filiform, 26-segmented, 1.3 times as long as body. Scape 1.5 times as long as its maximum width. First flagellar segment 4.3 times as long as its apical width, 1.1 times as long as second segment. Penultimate segment 2.5 times as long as wide, 0.5 times as long as first flagellar segment, and 0.9 times as long as apical segment; the apical segment shortly pointed apically.

Mesosoma. Length 1.8 times as long as high. Pronotum not convex dorsally (lateral view), with rather distinct pronotal carina. Median lobe of mesoscutum (dorsal view) not protruding forward, without median furrow. Notauli deep, narrow and distinctly crenulate with rugosity partly. Prescutellar depression rather deep, with three median carinae, entirely rugulose, 0.3 times as long as scutellum. Scutellum convex, without lateral carinae. Metanotum (dorsal view) with two strongly convergent and fused posteriorly lateral carinae and without median carinae; with distinct and pointed metanotal tooth. Subalar depression rather shallow, wide, rugose-reticulate. Sternaulus deep, straight, entirely smooth, connected with prepectal carina anteriorly, running along anterior 0.6 of the lower part of mesopleuron. Metapleural flange long, wide, rounded apically. Propodeum with very short, thick lateral tubercles.

Wings. Fore wing 3.0 times as long as its maximum width. Radial vein arising from middle of pterostigma. Radial cell not shortened. Metacarp 1.2 times as long as pterostigma. First radial abscissa 0.8 times as long as maximum width of pterostigma. Second radial abscissa 4.4 times as long as first abscissa, 0.7 times as long as the straight third abscissa and 1.3 times as long as first radiomedial vein. Second radiomedial cell 3.2 times as long as its maximum width, 2.4 times as long as brachial cell. First medial abscissa weakly curved. Mediocubital vein almost straight. Recurrent vein interstitial to first radiomedial vein. Distance from nervulus to basal vein almost equal to nervulus length. Parallel vein arising almost from middle of distal margin of brachial cell. Hind wing 5.0 times as long as its maximum width. First costal abscissa 0.6 times as long as second abscissa. First abscissa of mediocubital vein almost as long as second abscissa. Mediocubital cell large, distinctly widened toward apex, 7.5 times as long as wide, 0.4 times as long as whole length of wing. Recurrent vein straight, weakly antefurcal, unsclerotized.

Legs. Fore tibia with rather numerous slender spines arranged in almost single line. Hind coxa without dorsal tooth, 1.6 times as long as wide. Hind femur 3.6 times as long as wide. Hind tarsus 0.9 times as long as hind tibia. Hind basitarsus 0.6 times as long as second-fifth segments combined. Second segment of hind tarsus 0.6 times as long as basitarsus, 1.6 times as long as fifth segment (without pretarsus).

Metasoma 0.8 times as long as head and mesosoma combined. First tergite with distinct dorsope, without spiracular tubercles, rather weakly and almost linearly widened from base to apex. Maximum width of first tergite about twice its minimum width; length 1.1 times as long as its apical width. Second tergite without basomedian area, 0.7 times as long as its basal width, 1.2 times as long as length of third tergite. Second suture straight and shallow. Third tergite with shallow submedian transverse furrow. Ovipositor sheath 0.7 times as long as metasoma, 0.75 times as long as mesosoma and 0.3 times as long as forewing.

Sculpture and pubescence. Vertex and temple smooth; frons mostly smooth, finely granulate-coriaceous anterolaterally; face finely striate with dense fine granulation between striae, almost smooth laterally. Sides of pronotum entirely coarsely rugose-striate. Mesoscutum densely and coarsely granulate, coarsely rugose in narrow area on medioposterior half. Scutellum entirely densely granulate. Mesopleuron mostly smooth, finely granulate posteriorly. Propodeum with distinctly delineated by carinae areas; basolateral areas large, entirely coarsely granulate, additionally rugulose posteriorly; areola long and rather narrow, 1.7 times as long as wide; dorsal carina long, about 1.7 times as long as areola fork. Hind coxa dorsally coarsely rugose with granulation, densely granulate with fine rugosity laterally, almost smooth ventrally. Hind femur mostly smooth, finely rugulose-granulate dorsally. First tergite entirely densely, coarsely and linearly striate. Second tergite mostly rather finely and densely striate-rugulose, smooth posteriorly. Remaining tergites smooth. Vertex entirely with rather dense, short and semi-erect setae; mesoscutum entirely with dense, short and semi-erect pale setae. Hind tibia dorsally with rather long, very dense and semi-erect setae, length of these setae 0.6–0.8 times maximum width of hind tibia.

Colour. Body dark reddish brown with almost black spots, head brownish yellow below. Antennae brownish yellow in basal half, brown to dark brown in apical half. Palpi pale yellow. Legs yellow or brownish yellow, infuscate distally. Ovipositor sheath black. Fore wing membrane faintly infuscate. Pterostigma brown, yellow in basal 0.3 and apically.

Male unknown.

**Figure 2.**
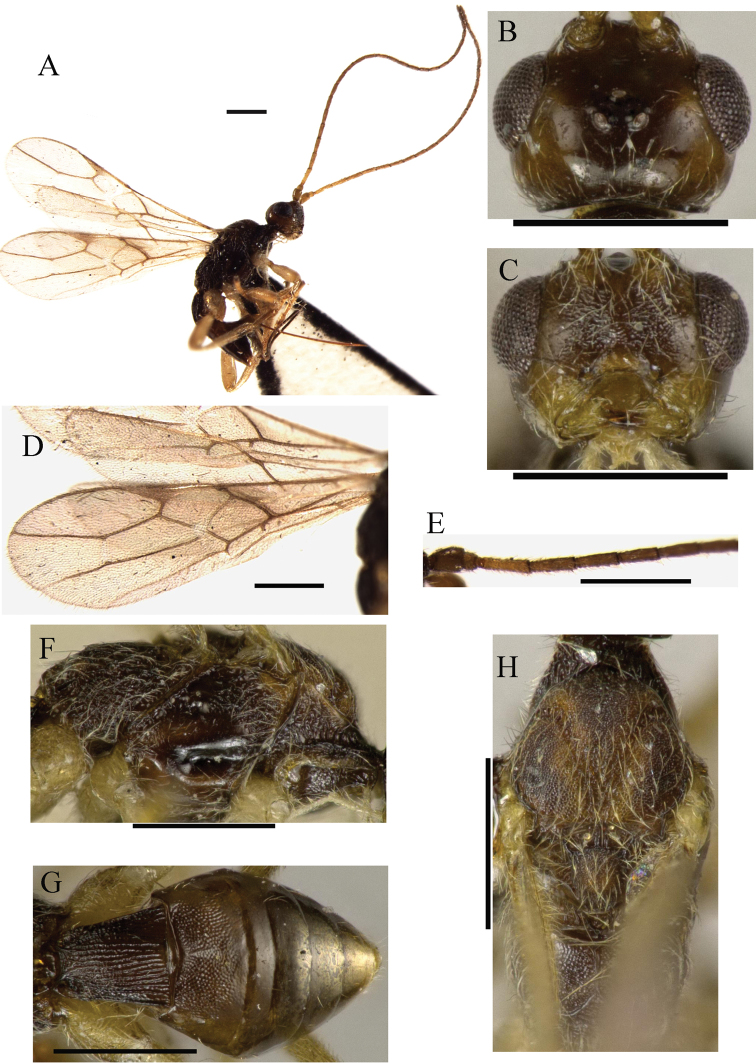
*Ontsira henana* sp. n. (female). **A** habitus, lateral view **B** head, dorsal view **C** head, front view **D** fore and hind wings **E** basal segments of antenna **F** mesosoma, lateral view **G** metasoma, dorsal view **H** mesosoma, dorsal view. Scale bar 0.5 mm.

##### Distribution.

China (Henan).

##### Diagnosis.

This new species is similar to *Ontsira eugeniae* Belokobylskij (Russian Far East), but differs in having the vertex and most part of frons smooth, the eyes glabrous, the second tergite widely sculptured, the antennal segments slender, the third tergite with transverse furrow, and the vertex and mesoscutum with short setae. It differs from *Ontsira gratia* Belokobylskij in having the second tergite widely sculptured, the second suture distinct, the third tergite with transverse submedian furrow, the hind femur wider, and the sternaulus smooth.

#### Ontsira ignea

(Ratzeburg, 1852)

http://species-id.net/wiki/Ontsira_ignea

Bracon igneus Ratzeburg, 1852: 36.
Doryctes igneus : Reinhard 1865: 250.
Doryctodes igneus : Telenga 1941: 91.
Ontsira ignea : Shenefelt and Marsh 1976: 1323; Belokobylskij and Tobias 1986: 43 (as synonym of *Ontsira imperator*); Belokobylskij and Maetô 2009: 370.

##### Material examined.

China: 1 female, Shaanxi, Ningshan, Huoditang, 1580 m, 17.VIII.1998 (Yuan Decheng) (ZJUH); 1 female, Fujian, Sangang, X.1979 (Huang Juchang), N 20003927 (ZJUH); 1 male, Guangdong, Meizhou, 29.VII.2003 (Chen Jujian), N 20048483 (ZJUH).

##### Distribution.

China (Shaanxi, Fujian, Guangdong) (**new record**); Japan Caucasus, Turkey, Israel, Central and Western Europe.

#### Ontsira imperator

(Haliday, 1836)

http://species-id.net/wiki/Ontsira_imperator

Rogas (Doryctes) imperator Haliday, 1836: 46; Yu et al. 2012.
Doryctodes imperator : Hellen, 1940: 26; Telenga 1941: 91.
Ontsira imperator : Marsh 1973: 71; Shenefelt and Marsh 1976: 1324; Belokobylskij and Tobias 1986: 43; Belokobylskij 1998a: 56; Chen and Shi 2004: 29; Yu et al. 2012; Belokobylskij and Maetô 2009.

##### Material examined.

China: 1 female, Jilin, Changchun, 8.X.1985 (Bai Hongyu), N 861610 (ZJUH).

##### Distribution.

China (Jilin, Taiwan); Japan, Korea, Mongolia, Russia, Kazakhstan, Iran, Caucasus, Central and Western Europe, North America.

#### Ontsira macer

Chen & Shi, 2000

http://species-id.net/wiki/Ontsira_macer

Ontsira macer Chen & Shi 2004: 30; Belokobylskij and Maetô 2009: 370.

##### Material examined.

China: 1 female, Zhejiang, Qingyuan, Baishanzu, 3.X.1993 (Wu Hong), N 945613 (ZJUH); 1 female, Zhejiang, Longquan, Fengyangshan, 29–31.VII.2007 (Liu Jingxian), N 200804390 (ZJUH); 1 female, Fujian, Wuyishan, 7.IX.1989 (Wang Jiashe), N 964392 (ZJUH); 1 female, Fujian, Jiangle, Longqishan, 16.VII.1991 (Liu Changming), N 20007069 (ZJUH); 1 female, Fujian, Erliping, XI.1979 (Huang Juchang), N 20003841 (ZJUH); 1 female, Guangdong, Shixing, Chebaling, 25.V.2002 (Xu Zaifu), N 20051526 (ZJUH).

##### Distribution.

China (Zhejiang, Fujian, Guangdong).

#### Ontsira neantica

Belokobylskij & Maetô, 2009

http://species-id.net/wiki/Ontsira_neantica

Ontsira neantica Belokobylskij & Maetô, 2009: 379.

##### Material examined.

China: 13 females, 5 males, China, Heilongjiang, Yichun, Daiceng, 29.V.1956 (Shi Zhenhua), N 5710.1(18) (ZJUH, ZISP).

##### Description of male

(first record). Body length 3.2–3.8 mm; fore wing length 3.0–3.4 mm. Transverse diameter of eye 0.9–1.0 times as long as temple. Frons mostly smooth. Antennae rather thick, 33-segmented. First flagellar segment 3.2–3.4 times as long as its apical width, 1.1–1.3 times as long as second segment. Prescutellar depression almost smooth, with three carinae. Second radial abscissa of fore wing 2.4–2.6 times as long as first abscissa, 0.4–0.5 times as long as third abscissa. Second radiomedial cell 2.2–2.4 times as long as its maximum width, 1.2–1.3 times as long as brachial cell. First abscissa of mediocubital vein of hind wing 1.2–1.4 times as long as second abscissa. Hind femur 3.3–3.6 times as long as wide. Metasoma 1.1–1.2 times as long as head and mesosoma combined.

##### Distribution.

China (Heilongjiang) (new record); Japan.

##### Remarks.

Vertex of females is usually striated on it sides.

#### Ontsira robusta sp. n.

http://zoobank.org/6266B6AA-1976-40FE-83EB-D59D4A208237

http://species-id.net/wiki/Ontsira_robusta

Fig. 3

##### Type material.

Holotype: female, “Korea, Kyonggi-do, Suwon-shi, Sodun-dong, Mt. Yogi, 23–29.VI.1994, Malaise trap” (ZISP).

Paratypes. 1 female, “Korea, Kyonggi-do, Suwon-shi, Sodun, Mt. Yogi, 16–23.VI.1994, Malaise trap” (ZISP); 1 female, China, North-East, 195(7?), N 5703.10 (ZJUH).

##### Etymology.

After Latin “robustus”, meaning “strong”, because of the first metasomal tergite short and wide.

##### Description.

Female. Body length 6.0–7.2 mm; fore wing length 4.8–5.4 mm.

Head width 1.2–1.3 times as long as its median length, about 1.1 times as long as width of mesoscutum. Frons without carina, with shallow or very shallow median furrow. Head behind eyes (dorsal view) weakly convex anteriorly, roundly narrowed posteriorly; transverse diameter of eye almost equal to temple. Ocellar triangle situated before middle of head (dorsal view), its anterior ocellus situated almost on middle level of eyes. Ocelli medium-sized, in triangle with base 1.2–1.3 times as long as its sides. POL 1.0–1.3 times Od, 0.4–0.5 times OOL. Eye rather densely and shortly setose, with very shallow or indistinct emargination opposite antennal sockets, 1.3–1.4 times as high as broad. Face along eyes without distinct carinae, with short and shallow elongate depressions above clypeus; width of face 1.15–1.20 times as long as height of eye and 1.3–1.4 times as long as height of face and clypeus combined. Diameter of antennal socket 1.1–1.4 times as long as distance between sockets and 1.4–1.6 times as long as distance between socket and eye. Malar suture indistinct. Malar space 0.40–0.45 times as long as height of eye, almost equal to basal width of mandible. Clypeus with wide flange ventrally. Clypeal suture complete, deep laterally and very shallow dorsally. Hypoclypeal depression round, its width 1.1–1.2 times as long as distance from edge of depression to eye, 0.45–0.50 times as long as width of face. Occipital carina ventrally fused with hypostomal carina at upper base of mandible. Maxillary palpi long, 1.1–1.2 times as long as head height.

Antennae rather thick, weakly setiform, 36–37-segmented, 0.75–0.80 times as long as body. Scape 1.7–1.9 times as long as its maximum width. First flagellar segment 2.7–3.0 times as long as its apical width, 1.15–1.30 times as long as second segment. Penultimate segment 1.7–1.9 times as long as wide, about 0.4 times as long as first flagellar segment, 0.7–0.8 times as long as apical segment; the apical segment distinctly pointed apically.

Mesosoma. Length about twice as long as its height. Pronotum weakly convex dorsally (lateral view), with fine pronotal carina in anterior 0.4. Median lobe of mesoscutum (dorsal view) rather distinctly protruding forward, with shallow and wide median furrow. Notauli deep anteriorly and shallow posteriorly, wide, densely coarsely crenulate-rugulose with granulation partly. Prescutellar depression rather deep, long, with one to three carinae, rather coarsely rugose entirely, about 0.5 times as long as scutellum. Scutellum weakly convex, without lateral carinae. Metanotum (dorsal view) with two strongly and curvedly convergent and fused posteriorly with large rugulose area lateral carinae, without median carinae; with short and obtuse metanotal tooth. Subalar depression shallow, wide, coarsely striate-rugose. Sternaulus deep, weakly curved or straight, oblique, densely and coarsely crenulate and with fine or very fine granulation, connected with prepectal carina anteriorly, running along anterior 0.6–0.7 of lower part of mesopleuron. Metapleural flange rather short, wide, rounded apically. Propodeum with rather distinct, short, thick lateral tubercles.

Wings. Fore wing 3.5–3.6 times as long as its maximum width. Radial vein arising behind middle of pterostigma, inner basal part of pterostigma 1.2–1.3 times as long as its inner apical part. Radial cell not shortened; metacarp 1.3 times as long as pterostigma. First radial abscissa 0.6–0.8 times as long as maximum width of pterostigma. Second radial abscissa 3.6–4.0 times as long as first abscissa, 0.6 times as long as the straight third abscissa, 1.3–1.5 times as long as first radiomedial vein. Second radiomedial cell 2.3–2.5 times as long as its maximum width, 1.1 times as long as brachial cell. First medial abscissa almost straight or weakly sinuate. Mediocubital vein weakly curved. Recurrent vein 3.0–3.3 times second abscissa of medial vein. Distance from nervulus to basal vein 0.7–1.0 times as long as nervulus. Parallel vein arising from posterior 0.25 of distal margin of brachial cell. Hind wing 4.7–5.2 times as long as its maximum width. First costal abscissa 0.6–0.7 times as long as second abscissa. First abscissa of mediocubital vein 1.5–1.8 times as long as second abscissa. Radial cell weakly narrowed posteriorly. Mediocubital cell large, widened toward apex, 6.5–6.7 times as long as wide, 0.45–0.50 times as long as whole length of wing. Recurrent vein straight or weakly curved, oblique, more or less distinctly postfurcal, unsclerotized but distinctly pigmented.

Legs. Fore tibia with numerous slender spines arranged in rather narrow stripe. Hind coxa without dorsal tooth, 1.3–1.5 times as long as wide (with tubercle). Hind femur 3.5–3.6 times as long as wide. Hind tarsus 0.85–0.90 times as long as hind tibia. Hind basitarsus 0.80–0.85 times as long as second-fifth segments combined. Second segment of hind tarsus 0.40–0.45 times as long as basitarsus, 1.20–1.25 times as long as fifth segment (without pretarsus).

Metasoma 1.1–1.2 times as long as head and mesosoma combined. First tergite with large dorsope, with small spiracular tubercles in basal 0.3, rather weakly and almost linearly widened from base to apex. Maximum width of first tergite 1.7 times its minimum width; length 0.9–1.1 times as long as its apical width. Second tergite without basomedian area, 0.5–0.6 times as long as its basal width, 0.85–0.90 times as long as third tergite. Second suture straight, shallow, complete. Third tergite without transverse furrow. Ovipositor sheath 0.8–1.1 times as long as body, 1.5–2.2 times as long as metasoma, 2.4–3.3 times as long as mesosoma, 1.1–1.4 times as long as fore wing.

Sculpture and pubescence. Vertex entirely or mostly more or less coarsely or (rarely) rather finely curvedly striate; frons entirely coarsely and partly undulately rugose-striate, with granulation between striae; face distinctly and rather densely transverse striate-rugose with dense and fine granulation, clypeus smooth with rugulosity below; temple rather coarsely or rather finely vertically striate, smooth on more or less narrow part near eye. Sides of pronotum entirely coarsely rugose, with crenulate median furrow. Mesoscutum densely and rather finely punctulate-rugulose entirely, with dense and rather fine granulation between rugae, coarsely rugose in wide area on medioposterior half. Scutellum mostly smooth. Mesopleuron rather coarsely or sometimes finely rugose, with two-three small smooth areas. Propodeum with rather distinctly delineated by carinae areas, entirely coarsely rugose-reticulate; basolateral areas large, entirely coarsely rugose-reticulate with dense granulation at least in anterior half; areola long and narrow, 1.6–1.8 times as long as wide; dorsal carina long, 1.1–1.7 times as long as areola fork. Hind coxae almost entirely and densely rugose-reticulate with dense and fine granulation between rugae. Hind femur rugulose-areolate with dense fine granulation dorsally and laterally, almost smooth ventrally. First tergite densely, coarsely linearly or curvedly striate, rugose-reticulate in mediobasal 0.3–0.7, without distinct ground sculpture between striae. Second tergite sparsely striate in basal 0.2–0.3, sometimes (holotype) with three semicircular striae or with curved striae basomedially, smooth on remaining part. Remaining tergites smooth. Vertex with rather sparse, short and semi-erect setae; mesoscutum entirely with very dense, short and semi-erect pale setae. Hind tibia dorsally with short, dense and almost erect or semi-erect setae, length of these setae about 0.5 times as long as maximum width of hind tibia.

Colour. Body black, metasoma behind first tergite dark reddish brown, yellowish brown or reddish brown ventrally, sometimes metasoma light reddish brown medially. Antennae dark reddish brown to black. Palpi reddish brown or sometimes dark reddish brown. Legs reddish brown, partly yellowish, all tibiae basally yellow. Ovipositor sheath black. Fore wing membrane rather distinctly infuscate. Pterostigma brown, sometimes very shortly paler basally and apically.

Male unknown.

**Figure 3.**
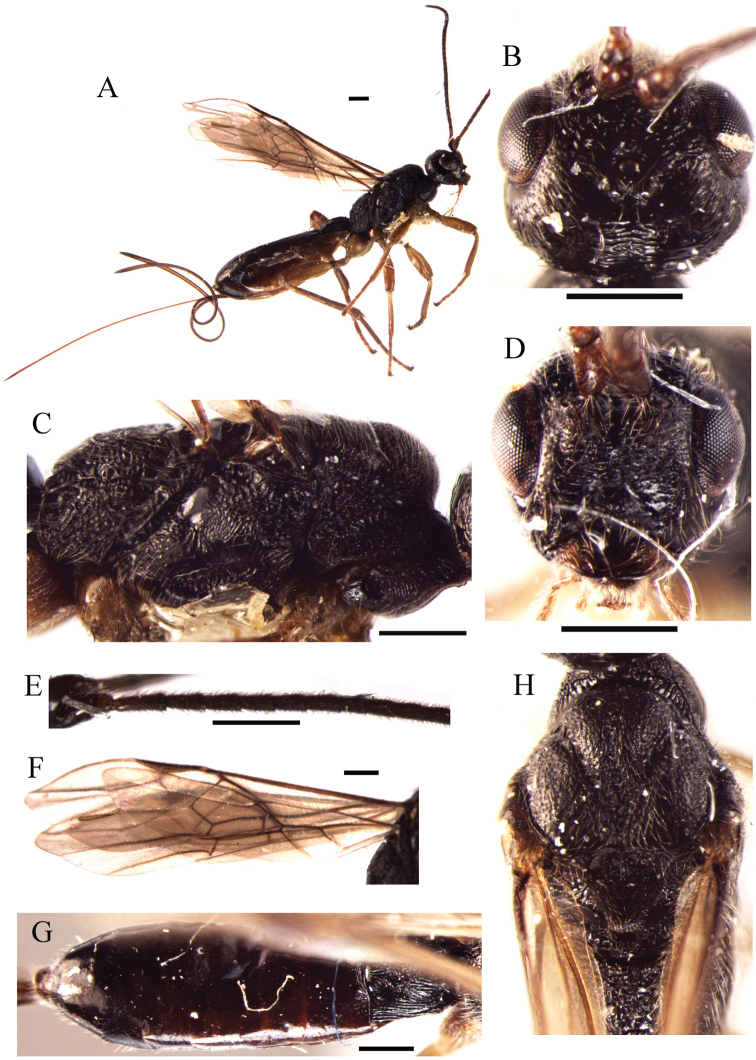
*Ontsira robusta* sp. n. (female). **A** habitus, lateral view **B** head, dorsal view **C** mesosoma, lateral view **D** head, front view **E** basal segments of antenna **F** fore and hind wings **G** metasoma, dorsal view **H** mesosoma, dorsal view. Scale bar 0.5 mm.

##### Distribution.

China (North East); Korea.

##### Diagnosis.

This new species is very similar to *Ontsira imperator* (Haliday), but differs in having the vertex almost entirely or mostly and temple at most part distinctly striate, and the mesoscutum almost entirely, very densely and finely granulate with sparse punctation. It is also similar to *Ontsira rugivertex* sp. n., but differs in having the antennae rather distinctly shorter than body, the first abscissa of mediocubital vein of hind wing longer, the hind femur wider, the second segment of hind tarsus shorter, the first tergite shorter and wider, the second tergite shorter, and the palpi dark.

#### Ontsira rugivertex sp. n.

http://zoobank.org/E349C77C-E5BB-4A7E-B1E0-3BF3B2AB5D88

http://species-id.net/wiki/Ontsira_rugivertex

Fig. 4

##### Type material.

Holotype: female, China, Henan, Neixiang, 14.VII.1998 (Chen Xuexin), N 988629 (ZJUH).

Paratype. China: 1 female, Shaanxi, Zhouzhi, Houzhenzi, 13.VI.1998 (Ma Yun), N 981419 (ZJUH).

##### Etymology.

After Latin “rugae” meaning “wringle” and “vertex” meaning “vertex, top” because of the vertex almost entirely and coarsely undulately striate.

##### Description.

Female. Body length 5.5–7.0 mm; fore wing length 5.0–6.2 mm.

Head width 1.2–1.3 times as long as its median length, 1.1 times as long as width of mesoscutum. Frons without carina, with shallow or very shallow median furrow. Head behind eyes (dorsal view) weakly convex anteriorly, roundly narrowed posteriorly; transverse diameter of eye 1.0–1.1 times as long as temple. Ocellar triangle situated distinctly behind middle of head (dorsal view), its anterior ocellus situated behind middle level of eyes. Ocelli medium-sized, in triangle with base 1.2–1.3 times as long as its sides. POL 1.0–1.2 times Od, 0.35–0.40 times OOL. Eye rather densely and shortly setose, with shallow or very shallow emargination opposite antennal sockets, 1.3 times as high as broad. Face along eyes without distinct carinae, with distinct small shallow elongate depressions above clypeus; width of face 1.2 times as long as height of eye and 1.20–1.25 times as long as height of face and clypeus combined. Diameter of antennal socket 1.0–1.3 times as long as distance between sockets and 1.5–1.7 times as long as distance between socket and eye. Malar suture indistinct. Malar space 0.45–0.50 times as long as height of eye and 0.8–0.9 times as long as basal width of mandible. Clypeus with wide flange ventrally. Clypeal suture distinct and complete. Hypoclypeal depression round, its width almost equal to distance from edge of depression to eye, 0.45 times as long as width of face. Occipital carina ventrall fused with hypostomal carina at upper base of mandible. Maxillary palpi long, 1.3–1.4 times as long as head height.

Antennae rather thick, almost filiform, 37–43-segmented, almost as long as body. Scape 1.8–2.0 times as long as its maximum width. First flagellar segment 3.6–3.8 times as long as its apical width, 1.2–1.3 times as long as second segment. Penultimate segment 2.0–2.2 times as long as wide, 0.4 times as long as first flagellar segment, 0.7 times as long as apical segment; the apical segment distinctly pointed apically.

Mesosoma. Length 1.8–1.9 times as long as high. Pronotum not convex dorsally (lateral view), with fine pronotal carina in posterior 0.4. Median lobe of mesoscutum (dorsal view) rather distinctly protruding forward, without or with shallow median furrow. Notauli deep, wide, densely coarsely crenulate and partly with rugosity. Prescutellar depression rather deep, without median carina, with several striae, coarsely rugose entirely, about 0.5 times as long as scutellum. Scutellum convex, without lateral carinae. Metanotum (dorsal view) with two strongly convergent and fused posteriorly lateral carinae, without median carinae; with short and obtuse metanotal tooth. Subalar depression shallow, wide, coarsely rugose-reticulate. Sternaulus deep, almost straight, densely and coarsely crenulate, connected with prepectal carina anteriorly, running along anterior 0.60–0.65 of the lower part of mesopleuron. Metapleural flange rather short, wide, rounded apically. Propodeum with distinct, short, thick lateral tubercles.

Wings. Fore wing 3.4 times as long as its maximum width. Radial vein arising weakly behind or from middle of pterostigma. Radial cell not shortened; metacarp 1.3–1.4 times as long as pterostigma. First radial abscissa 0.6–0.7 times as long as maximum width of pterostigma. Second radial abscissa 3.5–3.7 times as long as first abscissa, 0.5–0.6 times as long as the straight third abscissa, 1.3–1.5 times as long as first radiomedial vein. Second radiomedial cell 2.2 times as long as maximum width, 1.1–1.2 times as long as brachial cell. First medial abscissa almost straight. Mediocubital vein not curved. Recurrent vein 2.5–3.0 times as long as second abscissa of medial vein. Distance from nervulus to basal vein 0.4–1.0 times as long as nervulus. Parallel vein arising from posterior 0.15–0.25 of distal margin of brachial cell. Hind wing 4.5–4.8 times as long as its maximum width. First costal abscissa 0.7 times as long as second abscissa. First abscissa of mediocubital vein 1.2–1.5 times as long as second abscissa. Radial cell weakly narrowed posteriorly. Mediocubital cell large, distinctly widened toward apex, about 6.5 times as long as wide, 0.40–0.45 times as long as whole length of wing. Recurrent vein weakly and evenly curved, interstitial, sclerotized.

Legs. Fore tibia with rather numerous slender spines arranged in narrow stripe. Hind coxa without dorsal tooth, 1.6 times as long as wide. Hind femur 4.5 times as long as wide. Hind tarsus equal to hind tibia. Hind basitarsus 0.70–0.75 times as long as second-fifth segments combined. Second segment of hind tarsus 0.5 times as long as basitarsus, 1.6–1.8 times as long as fifth segment (without pretarsus).

Metasoma 1.1 times as long as head and mesosoma combined. First tergite with large dorsope, with small spiracular tubercles in basal quarter, rather weakly and almost linearly widened from base to apex. Maximum width of first tergite about twice as long as its minimum width; length 1.3–1.4 times as long as its apical width. Second tergite without basomedian area, 0.75–0.80 times as long as its basal width, 1.2 times as long as third tergite. Second suture straight, very shallow, almost absent medially. Third tergite without transverse furrow. Ovipositor sheath 1.1–1.2 times as long as body, 2.1–2.4 times as long as metasoma, 3.1–3.4 times as long as mesosoma, 1.2–1.4 times as long as fore wing.

Sculpture and pubescence. Vertex entirely or mostly more or less coarsely undulately striate, almost smooth near ocellar triangle; frons entirely coarsely rugose-reticulate; face distinctly and rather densely transverse striate, rugose medially, almost smooth below; temple coarsely rugose in upper 0.20–0.25, smooth on remaining part. Sides of pronotum entirely coarsely rugose-striate. Mesoscutum densely and distinctly rugose with dense fine granulation between rugae, coarsely and sparsely rugose in medioposterior half. Scutellum rather densely punctate, sometimes with very fine granulation between punctulae. Mesopleuron coarsely rugose-striate, rather narrowly smooth medially. Propodeum with distinctly delineated by carinae areas; basolateral areas large, entirely coarsely rugose-areolate; areola short and rather narrow, 1.4–1.5 times as long as wide; dorsal carina rather long, about twice as long as areola fork. Hind coxae almost entirely rugose-striate, partly with fine granulation, sculpture below fine. Hind femur dorsally and laterally rugose with fine granulation, almost smooth ventrally. First tergite densely, coarsely and linearly striate with fine rugulosity between striae. Second tergite densely striate in basal 0.3. Remaining tergites smooth. Vertex with rather dense, short and semi-erect setae; mesoscutum entirely with dense, short and semi-erect pale setae. Hind tibia dorsally with short, very dense and semi-erect setae, length of these setae about 0.5 times as long as maximum width of hind tibia.

Colour. Body black; metasoma medially or behind first tergite reddish brown. Antennae black, dark reddish brown basally. Palpi pale yellow. Legs light reddish brown, all coxae, trochanters and tibiae basally yellow, the remaining tibiae and tarsi sometimes reddish brown to dark reddish brown. Ovipositor sheath black. Fore wing membrane faintly infuscate. Pterostigma brown, shortly paler basally and apically.

Male unknown.

**Figure 4.**
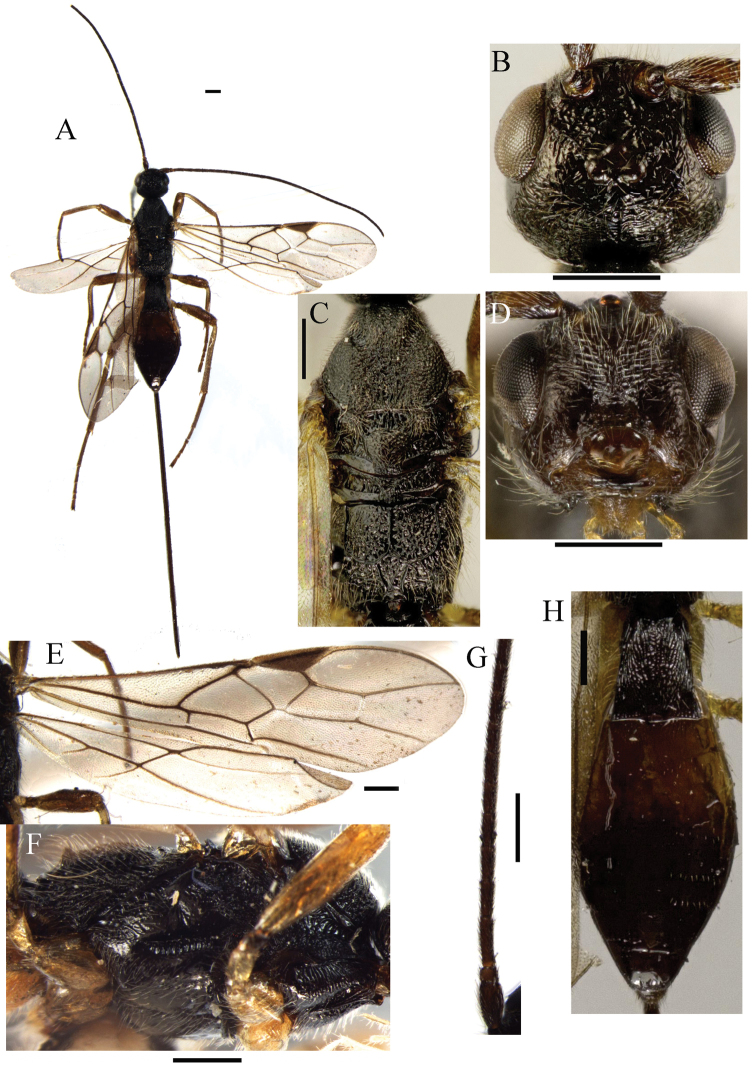
*Ontsira rugivertex* sp. n. (female). **A** habitus, dorsal view **B** head, dorsal view **C** mesosoma, dorsal view **D** head, front view **E** fore and hind wings **F** mesosoma, dorsal view **G** basal segments of antenna **H** metasoma, dorsal view. Scale bar 0.5 mm.

##### Distribution.

China (Henan, Shaanxi).

##### Diagnosis.

The new species is similar to *Ontsira ignea* Ratzeburg, but differs in having the vertex coarsely rugose-striate entirely or almost entirely, the first flagellar segment shorter, the second tarsal segment of hind leg distinctly longer than fifth segment (without pretarsus), and the mesoscutum coarsely rugose-granulate.It resembles *Ontsira robusta* sp. n., but their differences are listed after the description of *Ontsira robusta*.

## Supplementary Material

XML Treatment for Ontsira

XML Treatment for Ontsira abbreviata

XML Treatment for Ontsira antica

XML Treatment for Ontsira apposita

XML Treatment for Ontsira henana

XML Treatment for Ontsira ignea

XML Treatment for Ontsira imperator

XML Treatment for Ontsira macer

XML Treatment for Ontsira neantica

XML Treatment for Ontsira robusta

XML Treatment for Ontsira rugivertex
